# Precision cut lung slices: an innovative tool for lung transplant research

**DOI:** 10.3389/fimmu.2024.1504421

**Published:** 2024-11-28

**Authors:** Denny Joseph Manual Kollareth, Ashish K. Sharma

**Affiliations:** ^1^ Department of Surgery, University of Florida, Gainesville, FL, United States; ^2^ Division of Pulmonary and Critical Care Medicine, Department of Medicine, University of Florida, Gainesville, FL, United States; ^3^ Department of Pharmacology and Therapeutics, University of Florida, Gainesville, FL, United States

**Keywords:** precision cut lung slices, lung ischemia reperfusion injury, lung transplantation, primary graft dysfunction, inflammation, oxidative stress

## Abstract

Lung ischemia-reperfusion injury (IRI), a common complication after lung transplantation (LTx), plays a crucial role in both primary graft dysfunction (PGD) and chronic lung allograft dysfunction (CLAD) thereby adversely impacting the clinical outcomes in these patient cohorts. Lung IRI is characterized by several molecular events including immune cell infiltration, reactive oxygen species (ROS) generation, calcium overload, inflammation and various forms of cell death pathways. Currently, no therapeutic agents are available to clinically prevent lung IRI. While animal and cell culture models are highly valuable in understanding the pathophysiology of lung IRI, they may not completely recapitulate the complexity of human lung tissue pathology. This limitation necessitates the requirement for developing innovative preclinical human research tools that can supplement available scientific modalities. Emerging evidence suggests that precision-cut lung slices (PCLS) have become an indispensable tool in scientific research to study lung biology in an *ex vivo* tissue system. Recent studies using PCLS have investigated lung diseases including asthma, chronic obstructive pulmonary disease (COPD), and lung cancer. Although PCLS can be successfully employed to determine the deleterious events in the pathogenesis of lung IRI, including cell-cell interactions as well as hallmarks of inflammation and oxidative stress-dependent pathways, detailed studies employing PCLS to decipher these molecular events in post-LTx injury are currently limited. This review focuses on the applicability and unexplored potential of PCLS as a powerful tool in lung IRI research for understanding the pathophysiology and consequent development of new therapeutic modalities.

## 1 Introduction

Respiratory diseases rank among the leading causes of morbidity and mortality worldwide, and the extremely high disability rate imposes a huge financial burden on the families of patients (1). As they affect millions worldwide, these conditions are critical areas of medical research. Lung diseases can be categorized broadly into chronic and acute. Chronic respiratory diseases are a group of different conditions affecting the airways, which all impair lung function over time. These include asthma and rhinitis, chronic obstructive pulmonary disease (COPD), occupational lung diseases, sleep-disordered breathing (SDB), pulmonary hypertension and pulmonary interstitial diseases (2). Acute respiratory diseases are conditions that affect the respiratory system (airways, lungs) and have a sudden onset, typically lasting a short period (a few days to a few weeks). These include acute lung injury (ALI), acute respiratory distress syndrome (ARDS) and COVID-19-associated ARDS (3). Lung ischemia-reperfusion injury (IRI) is a form of acute lung injury (ALI), commonly occurring after lung transplantation (LTx) (4). In-depth molecular and cellular signaling pathway analysis of allograft tolerance or rejection requires reliable and reproducible *in vitro* and *in vivo* models of primary graft dysfunction (PGD) and chronic lung allograft dysfunction (CLAD). Recent advances in *ex vivo* models include precision cut lung slices (PCLS) that represent uniform tissue slices generated from animal or human lungs, and have proven to be a valuable tool to facilitate high-throughput studies in basic lung biology (5). In this review, we explore the applications of PCLS in lung IRI, highlighting the importance and utility of PCLS as a valuable tool in investigating post-LTx injury.

## 2 Lung ischemia reperfusion injury

LTx is a potentially life-prolonging therapy for patients with end-stage lung disease; however, potential benefits are limited due to several complications that result in chronic rejection of the allograft tissue (6). Despite advances in surgical management and immunosuppression, survival rates following LTx remain significantly lower compared to other solid organ transplants, with a current median survival of 5.8 years (7). While restoring blood flow is essential for recovery, reperfusion itself causes further damage, triggering acute, sterile inflammation. IRI is therefore defined by two phases of organ damage: the initial phase, caused by the interruption of blood supply (during organ procurement), and the second phase, occurring when blood flow is restored (reperfusion phase during post-LTx) (8). Clinically, lung IRI can lead to pulmonary dysfunction, decreased PO_2_, edema, increased pulmonary vascular resistance and enhanced vascular permeability causing immune cell infiltration and inflammation. Lung tissue oxygenation is compromised as ventilation/perfusion (V/Q) mismatch occurs due to pulmonary edema and elevated peak airway pressures that leads to inadequate ventilation (9).

At the cellular level, lung IRI is characterized by exacerbation of cellular dysfunction and cell death. During IRI, a rapid accumulation of reactive oxygen species (ROS) occurs shortly after reperfusion, accompanied by increased activity of ROS-generating enzymes (10). Intracellular calcium overload is another major event in the initial steps of lung IRI (11). Calcium overload and excessive ROS generation trigger both apoptosis and necrosis causing a further release of ROS and proinflammatory cytokines. These multifaceted processes activate resident lung macrophages, an early and critical source of pro-inflammatory mediators that orchestrate the IRI response involving immune as well as parenchymal cells such as alveolar type II (ATII) epithelial cells (12). Furthermore, chemokine secretion, such as C-X-C motif chemokine ligand 1 (CXCL1) by ATII cells, and increased endothelial permeability leads to infiltration of neutrophils, which plays an important role in pathophysiology of lung IRI (13). These inflammatory pathways and mechanisms contribute to increased pulmonary vascular resistance and microvascular permeability, endothelial cell dysfunction, resulting in pulmonary edema and impaired gas exchange, that are established clinical hallmarks of PGD (12, 14). Emerging evidence also shows that IRI induced tissue/cell injury involves regulated cell death pathways such as autophagy, apoptosis, necroptosis, and ferroptosis (15). Taken together, the inflammatory milieu in the post-LTx tissue microenvironment involves a synchronized crosstalk between immune cells such as alveolar macrophages and neutrophils, as well as parenchymal cells such as ATII cells and endothelial cells, which culminate in IRI thereby leading to acute rejection.

### 2.1 Models of lung IRI

Preclinical models are essential to address knowledge gaps in three of the most vexing and serious LTx complications: PGD, acute rejection (AR), and CLAD. Traditional animal models and *ex vivo/in vitro* models are the common experimental models used in studying lung IRI.

#### 2.1.1 *In vivo* models

Orthotopic LTx, which is more commonly performed using the left lung, has been used to study PGD and recapitulate the various steps in clinical LTx such as cold and warm ischemia as well as immunosuppression using murine models (16–18). This model has the advantage that it simulates the surgical procedure of human LTx. Orthotopic LTx is a technically demanding procedure but is more ideal for studying immune response following LTx (19). Researchers have explored various anti-inflammatory and immune regulatory therapies targeting different pathways in this small animal LTx model (20, 21).

Another commonly used model to study lung IRI is the hilar ligation model (21). This is an efficient, accelerated proxy for lung IRI, eliciting the same pathophysiological changes in a shorter timeframe that recapitulates the preclinical framework of ALI (22). This murine model involves mechanical ventilation, a thoracotomy to expose the left lung hilum, and occlusion of all hilar structures with a microvascular clamp for a variable period of warm ischemia followed by unclamping allowing for reperfusion (23). This model showed extensive pulmonary tissue injury evident by increased neutrophil infiltration, apoptosis and pulmonary dysfunction (21, 22, 24). Another variation of the hilar clamp model involves isolating and clamping only the pulmonary artery while preserving gas exchange, known as the nonhypoxic lung ischemia model (25). An advantage of the hilar ligation model for studying IRI component of PGD is that it is less technically challenging than orthotopic single LTx models, but the limitations include lack of recapitulation of cold storage, donor-dependent characteristics, and absence of immunosuppression. Overall, these murine models make them an appropriate platform for testing therapeutic interventions, evaluating rehabilitative potential, and identifying biomarkers predictive of PGD (21, 23, 24, 26).

#### 2.1.2 *Ex vivo* lung perfusion

*Ex vivo* lung perfusion (EVLP) has developed as a novel method to maintain donation after cardiac death (DCD) lungs in physiologically protective conditions outside the body and allows accurate evaluation of lung function, as well as providing a new platform for therapeutic treatment and repair of damaged donor lungs before transplantation (27). During EVLP, the donor lungs are subjected to a perfusion apparatus that offers a controlled environment, allowing for the delivery of oxygen and nutrients while removing carbon dioxide and other waste products. EVLP has been used to study several molecular events and therapeutic agents in lung IRI, including interleukin-10 (IL-10), mesenchymal stem cell-derived extracellular vesicles, β-adrenoreceptor agonists, and sevoflurane, yielding promising results in preconditioning of donor lungs to increase the donor pool and with the goal to mitigate post-LTx IRI (14). Several preclinical studies using EVLP in porcine LTx models have shown the relevance of temperature regulation of donor organ preservation with normothermic and hypothermic conditions to enhance the suitability of DCD organs and attenuate post-LTx IRI, that can be effectively studied using this technique (28–33).

#### 2.1.3 *In vitro* models

Detailed cellular level analysis facilitated by *in vitro* models of tissue injury and induction of IRI have been successfully reported in cell lines of kidney, liver, heart and lung (34). To closely mimic the process of ischemia and subsequent reperfusion, previous studies have used *in vitro* hypoxia-reoxygenation (HR) as a model in lung cells. In this model, cells are cultured in a controlled environment with reduced oxygen levels (typically 1–5% O_2_) or in the complete absence of oxygen. After the hypoxia period, the cells are re-exposed to normal oxygen levels (20–21% O_2_) to simulate reperfusion that lead to substantial production of oxidative stress and inflammation (21, 35, 36). The use of co-cultures and transwell membranes using different cell types also facilitates cell-cell interaction that is a key hallmark of lung IRI.

While animal models have significantly advanced our understanding of disease mechanisms and are crucial for studying *in vivo* processes, they do not necessarily mimic the nature and complexity of native human tissue and genome. Additionally, in some cases, such as with species-specific infections, especially in pulmonary diseases, animals may not entirely recapitulate the immunogenic response of human disease. Moreover, animal models are also limited to provide high-throughput analysis of drugs or pathogen isolates (37, 38). Furthermore, *in vitro* models encompassing cells in culture may not accurately simulate the *in vivo* environment experienced by lung cells during transplantation. Since the cell culture systems do not replicate the complex structure of pulmonary tissue, the ability of *in vitro* test results has inherent limitations as well (39, 40). Hence, there is a need for improved, scalable models, which also will enable reduction and refinement of animal research (37). Thus, recent evidence suggests that PCLS has emerged as a highly relevant *ex vivo* model for studying injury and repair responses, as well as for the development of novel therapies.

## 3 Precision cut lung slices

PCLS are thin and uniform lung slices made from human or animal lung tissues that have attracted increasing attention (1). PCLS can be prepared from naïve, genetically modified, or diseased animals as well as from human surgical samples and lung explants, and can be even cryopreserved for future use (41). The *ex vivo* tissue obtained from PCLS maintains the homogeneity and architecture of the primary tissue and retains resident cell content and morphology of the lung, including smooth muscle cells, epithelial cells, and fibroblasts. These cells maintain their intercellular interactions and cell-to-matrix relationships within the complex structures of the lung. PCLS are now widely utilized to assess integrated cellular responses triggered by inflammatory, fibrotic, or infectious stimuli, as well as for investigating lung damage and regeneration (42). Studies have compared *in vivo* exposed lungs and PCLS behavior and sensitivity to a wide panel of stimuli (physical, mechanical, chemical, pharmacological). Collectively, these studies conclude and suggest that PCLS respond in a manner similar to *in vivo* lung tissue (43). The development of PCLS technique, with improved methods for maintaining viability and function in prolonged culture, has resulted in numerous applications in pulmonary research. PCLS have been used in lung anatomy studies also been used in numerous toxicology studies to assess the efficacy and safety of new therapeutic targets (44). Further, PCLS have proven to be useful for studying a variety of conditions including asthma, allergic responses, vascular responses, early fibrosis, COPD, alveologenesis, lung cancer and respiratory infections, among other applications (37, 43, 44).

### 3.1 Preparation and maintenance of PCLS

The history of PCLS can be traced back to the general development of techniques for preparing and studying tissue slices in general. The development of tissue-slicing techniques began in the late 19th and early 20th centuries when researchers used specialized instruments for cutting thin sections from various tissues for microscopic examination (45). The development of vibratome/microtome allowed for the production of tissue slices with greater precision and consistent thickness (46). A significant breakthrough in PCLS was achieved in 1980s, where in Placke and Fisher infused airways of rat and hamster lungs with heated liquid agarose. This agarose solidifies at temperatures below 25°C, helps in maintaining the inflated state of the lung and prevents the collapse of the airways and delicate alveoli during the slicing process (47). This method was further developed and first applied to human lung tissue in 1994 (48). Since then, various adaptations have been made and detailed step‐by‐step protocols for generating PCLS from human and mouse tissue have been reported in the literature (49, 50).

**Figure 1:**
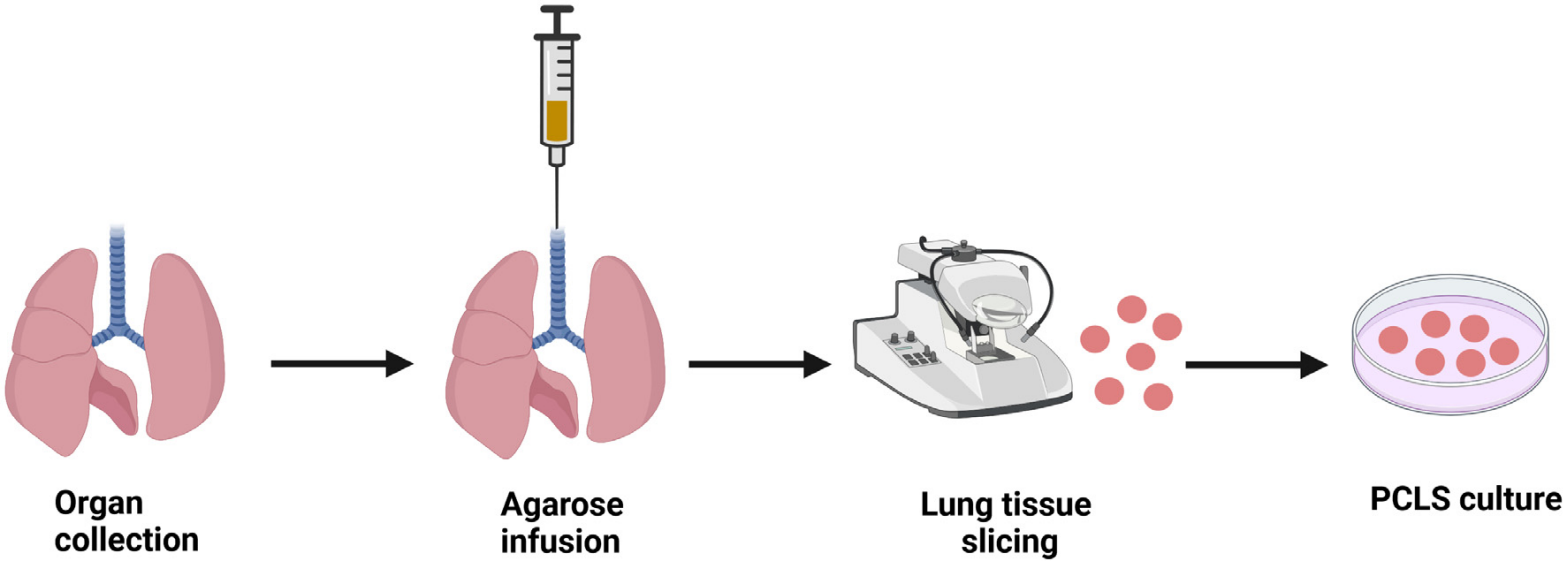
Schematic diagram illustrating the procedure to generate precision cut lung slices (PCLS). Lung tissue is harvested and PCLS is prepared using a specialized tissue slicer following agarose infusion into the lungs. *Ex vivo* PCLS can be then cultured in appropriate conditions for studying respective pathological milieu. Image created using biorender.com.

Central to all protocols in the preparation of PCLS is that the freshly retrieved whole lungs or individual lobes are infused with warm, low-melting agarose dissolved in buffer or tissue culture medium, at a concentration ranging from 0.5% to 3% (38) (**Figure 1**). The most common and easiest way to deliver the agarose to the lung is by cannulating the trachea in small animals (42). In human PCLS, lung sections are filled via the main bronchus with low melting point agarose solution (51). However, human lung samples are often received as partial lobes from surgical resections. If the airways cannot be identified, multiple small injections of agarose with a fine needle can be made directly into the alveolar tissue to inflate the lung (52). The lungs are then cooled to below 25°C by immersing the tissue in cold buffer to solidify the agarose and stiffen the parenchyma before slicing. After solidification, the tissue is sliced using a vibratome/compresstome to obtain PCLS of uniform thickness between 150-500 µm (38). Protocols for culturing PCLS vary in terms of culture media conditions and duration, depending on the specific outcomes being studied. Serum is either absent or added in very low quantities to prevent cell proliferation and changes to the native cellular diversity (52). For incubation, 50 µg/mL gentamicin and 0.25 µg/mL amphotericin B are added to serum-free minimal essential medium (MEM) and then placed in a 5% CO_2_ incubator at 37°C. The medium is changed every 30 min for the first 2 hours, once per hour for the next 2 hours, and then switched to 24 hour intervals to remove the residual agarose and cell debris (1). Most studies using PCLS have been conducted under short-term culturing conditions (≤72 h), but there are reports showing that PCLS can remain viable for up to 28 days (53).

### 3.2 Application of PCLS in LTx research

The number of studies utilizing PCLS in LTx research is limited (54, 55). Hence, the application of PCLS in lung IRI is an underexplored area that could play a significant role in advancing LTx research. PCLS can be used to investigate many of the pathological events during lung IRI, making it a powerful tool for studying this condition. Here, we describe the deleterious molecular events in lung IRI that can be effectively studied by employing PCLS.

#### 3.2.1 PCLS as a research tool for oxidative stress and inflammation

The robust oxidative stress and sterile inflammation occurring upon reperfusion is a key driver of PGD leading to inferior short- and long-term survival in LTx (4, 12). Baste et al. (54) demonstrated that PCLS can be used to study lung IRI by employing a hybrid model, with an *in vivo* ischemic period followed by *ex vivo* reoxygenation (54). In this study, following *in vivo* ischemia, lungs excised, PCLS were prepared and transferred to medium for *ex vivo* reoxygenation. This study indicated that there was induction of oxidative stress, as evidenced by changes in antioxidant enzyme activities, representing the ability of PCLS tissue to mimic a pivotal cellular process of lung IRI. Histological analysis indicated bronchiolar epithelial hyperplasia and necrotic changes in the lung slices, mainly after longer ischemic durations, suggesting the presence of inflammatory responses in this pulmonary tissue (54, 56). In a recent study, PCLS prepared from human or porcine lungs were used to model acute rejection (55). Peripheral blood mononuclear cells, isolated from blood type-matched donors to mimic transplantation, were introduced into the tissue to measure immune responses. The results showed that PCLS exhibited histological signs of acute rejection, such as increased immune infiltration and alveolar wall thickening (55). In another study, PCLS were employed to detect changes in cytokine levels, oxidative stress markers and mitochondrial activity in response to nanomaterials (40). The study found increased levels of cytokines in response to a number of nanomaterials (tumor necrosis factor-alpha (TNF-α), Interleukin-8 (IL-8), macrophage colony-stimulating factor (M-CSF), and osteopontin (OPN)). These cytokines reflect different stages and aspects of inflammation, indicating the model’s ability to capture inflammatory responses. The study also showed significant alterations in reduced glutathione (GSH) levels, indicating induction of oxidative stress and confirming that PCLS can be used to determine inflammation and oxidative stress due to nanomaterial toxicity (40). Similarly, PCLS were able to produce pro-inflammatory cytokines upon lipopolysaccharide (LPS) stimulation. Cultured PCLS were also shown to be capable of generating re-call immune responses, characterized by cytokine production, against antigens commonly found in routine vaccinations against influenza virus and tetanus toxoid. This suggests that PCLS have the potential to exhibit immunologic memory, allowing them to respond to previously encountered antigens (57). In another study employing human PCLS, the initial response to LPS exposure was marked by a coordinated release of early release cytokines such as interleukin-1 beta (IL-1β), macrophage inflammatory protein-1 beta (MIP-1β), and Interferon-gamma (IFN-γ). These cytokines are produced mainly by macrophages, epithelial cells, fibroblasts, and/or endothelial cells, and they play a significant role in activating epithelial or airway smooth muscle cells. Interestingly, the study also showed that cytokine production in human lung slices and human BAL fluid from *in vivo* studies after treatment with LPS are correlated (58), reinforcing the applicability of PCLS in investigating the molecular pathways of pulmonary inflammation. PCLS has also been used to study the behavior and activation of macrophages in the lung environment (59). The use of PCLS as an *ex vivo* model to delineate lung tissue inflammatory responses in respiratory diseases has been reported recently (1).

Endothelial cell dysfunction and disruption of the endothelial barrier are hallmarks of lung IRI and contribute greatly to PGD. Depolarization of endothelial cell membranes induces ROS production and subsequent inflammation (4). PCLS has been employed to study the effects of cigarette smoke on endothelial functions. The findings showed impaired vasorelaxation and increased sensitivity to vasoconstriction, which are indicative of endothelial dysfunction (60). In a recent study, PCLS exposed to nitrogen mustard (a cytotoxic agent), showed evidence of oxidative stress as shown by reduced total and oxidized glutathione levels and increased catalase levels (61). Further, oxidative stress was elevated in PCLS exposed gasoline exhaust particles (62). PCLS exposed to organophosphorus pesticides exhibited reduced GSH levels and increased oxidized glutathione levels (63). Furthermore, PCLS subjected to hypoxia exhibited increased ROS levels (64). Altogether, PCLS represents a valuable tool for investigating inflammation and oxidative stress, the major deleterious molecular events occurring during IRI, making it a useful model for studying IRI mechanisms and testing various therapeutic modalities.

#### 3.2.2 Application of PCLS to measure calcium overload

Intracellular calcium overload is one of the main pathological mechanism of lung IRI (65). A recent study has shown that PCLS can be used to evaluate intracellular calcium signaling (66). Employing Oregon green to measure intracellular Ca^2+^ levels in lung slices, a previous study showed that Ca^2+^ oscillations are essential for maintaining the contractile state of the airway smooth muscle cells and, thus, the airway contraction (67). It is shown that the inhibition of calcium activated potassium channels increased cytoplasmic Ca^2+^ in PCLS (68). Furthermore, the correlation of airway contraction with smooth muscle cell Ca^2+^ signaling has been possible by combining the PCLS with real-time confocal or two-photon microscopy (67, 69). These reports suggest the applicability of using *ex vivo* PCLS to decipher the relevance of calcium and ion-mediated signaling in post-LTx IRI.

#### 3.2.3 Application of PCLS to study cell death pathways

Various cell death pathways have been observed in lung IRI, including apoptosis, necrosis, pyroptosis and ferroptosis (70). PCLS treated with hyperoxia exhibited increased DNA fragmentation, a hallmark sign of apoptosis and necrosis as revealed by TdT-mediated dUTP-biotin nick end labeling (TUNEL) staining (71). Furthermore, PCLS treated with nanoparticles exhibited increased apoptosis indicated by significant increase in caspase-3 staining (72, 73). In another study, whole diesel exhaust induced a pro- inflammatory and apoptotic response in PCLS which were reduced by exhaust filtration (74). A recent study has shown that PCLS can be used to investigate ferroptosis (75). Together, these studies suggest that PCLS can be effectively employed to delineate various cell death pathways that have been reported in post-LTx injury.

## 4 Summary, limitations and future perspectives

### 4.1 Summary

**Figure 2:**
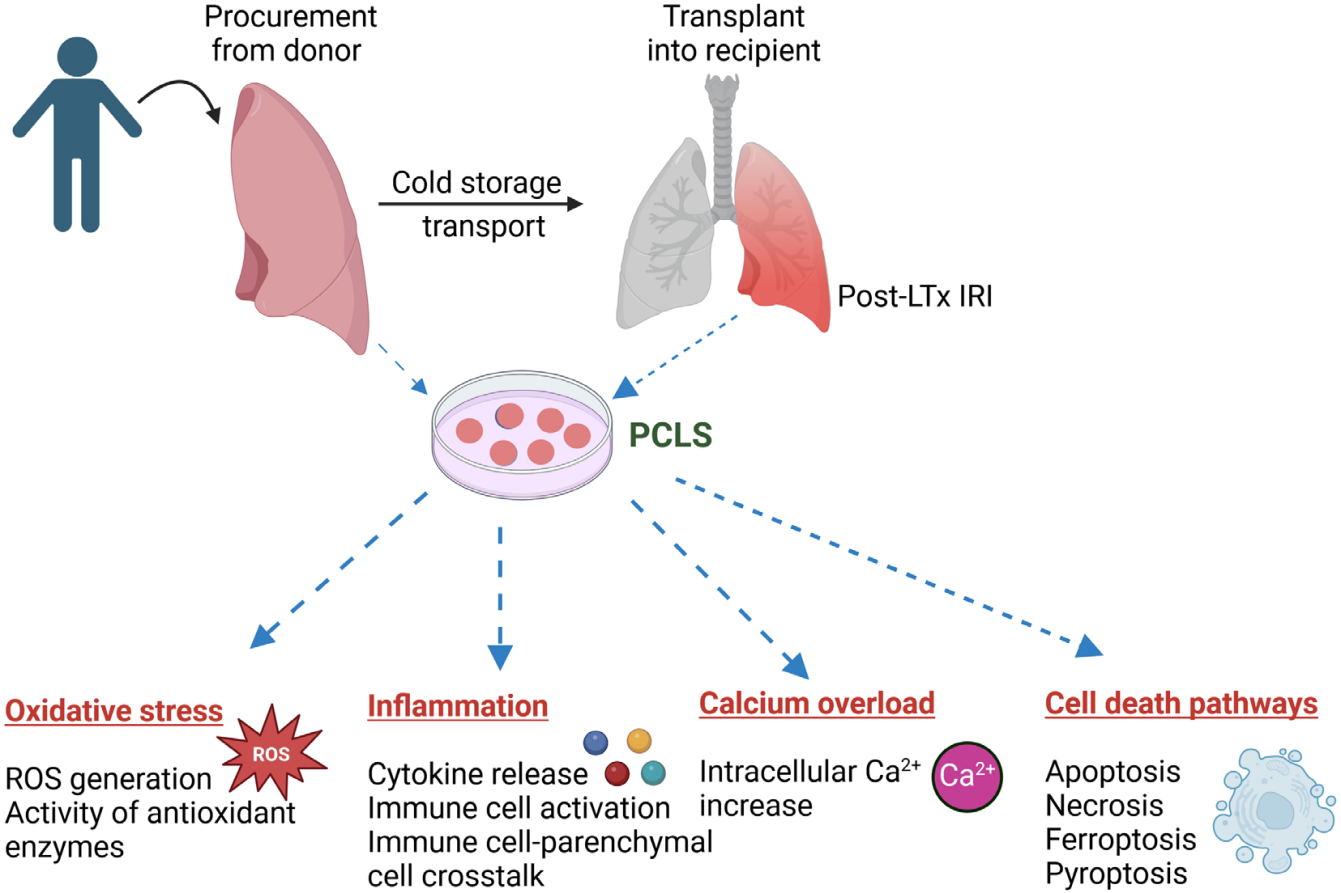
Application of precision cut lung slices (PCLS) in investigating the pathogenesis of lung ischemia reperfusion injury (IRI) following lung transplantation (LTx). PCLS tissue can be generated from donor or recipient lungs to study a myriad of biological processes, such as oxidative stress, inflammation, Ca^2+^ overload and various cell death pathways, involved in lung IRI. Image created using biorender.com.

Lung IRI is the main risk factor for primary graft dysfunction and mortality following LTx. While extensive research has been conducted on lung IRI using other established animal and cell culture models, the underlying mechanisms and signal transduction pathways remain elusive, and a specific, effective treatment is still unavailable. PCLS can make a huge advancement in lung IRI research by bridging the gap between *in vitro* and *in vivo* models. PCLS reflects the complex cellular composition and the matrix effects of a diseased human lung, and biological diversity underlying differential treatment response in patients may well be studied in PCLS from different patients (76). The integration of agarose stabilization, precise mechanical cutting, and advanced imaging techniques has transformed PCLS into a powerful tool for studying lung physiology in both health and disease (52). Although many of the molecular events associated with lung IRI, such as oxidative stress, inflammation, calcium overload, and cell death pathways, can be studied using PCLS, only a few studies have employed this model so far. Recent evidence and previous studies, as summarized above, demonstrate that PCLS can be a valuable tool for investigating the pathogenesis and delineating the mechanistic potential of therapeutic targets in the treatment of lung IRI (**Figure 2**). Thus, PCLS offers a robust, cutting-edge platform that can drive significant advances in the study and treatment of lung IRI.

### 4.2 Limitations of using PCLS in LTx research

Several potential tissue-related challenges arise when using PCLS such as cellular changes induced by agarose instillation into the lung and pre-contraction of airways (51). Issues with agarose instillation can result in problems such as mushy lungs when cutting sections and shredded tissue (77). Further, the vast amount of agarose in PCLS impedes RNA extraction by standard procedures (78). Additionally, PCLS currently lack a standardized cell culture medium, and an optimal method for medium optimization has yet to be established (1). Further, human lung tissue suitable for slicing is not available at many research institutions, and obtaining such tissue is challenging due to varying clinical and regulatory frameworks (37). Additionally, the effects of breathing and air-liquid interface cannot be studied employing PCLS (76). Furthermore, PCLS are isolated from the circulatory system in the body, making it difficult for researchers to evaluate the migration of cells between the lung, blood, and lymph nodes and preventing the replication of cellular trafficking of inflammatory cells and humoral factors, as well as antigen presentation that primes the immunogenic response in the *in vivo* environment. These limitations dampen the ability to evaluate adaptive immune responses and therefore needs further refining of this technique (1, 79).

### 4.3 Future perspectives

PCLS offers a versatile platform for investigating a wide range of lung diseases. However, optimizing and refining PCLS methods is essential to unlock its full potential as a research tool. Enhancing the longevity, structural integrity, and functional relevance of PCLS is essential to develop it into a model that more accurately reflects *in vivo* conditions. The short duration of PCLS culture viability is a major drawback in the field. Although attempts have been made to extend it, further attention is needed to improve both the longevity and functionality of PCLS. Additionally, PCLS should be combined with advanced imaging techniques to visualize three-dimensional cell-cell interactions, overcoming the limitations of two-dimensional imaging. Genetic manipulation or omics approaches should be applied to PCLS to gain deeper understanding of lung health and disease. As discussed in this review, there is significant potential for employing PCLS in LTx research. Likewise, the use of PCLS in lung research should be expanded to study complex lung diseases and drug responses that remain unexplored. In summary, utilizing PCLS to further our understanding of molecular events and explore various therapeutic options for post-LTx lung IRI represents a promising avenue for future research in this field. By bridging gaps between *in vitro* studies and clinical applications, PCLS present valuable potential for translational advancements in pulmonary research.
